# TiS_2_ as an Advanced Conversion Electrode for Sodium‐Ion Batteries with Ultra‐High Capacity and Long‐Cycle Life

**DOI:** 10.1002/advs.201801021

**Published:** 2018-09-15

**Authors:** Hongwei Tao, Min Zhou, Ruxing Wang, Kangli Wang, Shijie Cheng, Kai Jiang

**Affiliations:** ^1^ State Key Laboratory of Advanced Electromagnetic Engineering and Technology School of Electrical and Electronic Engineering Huazhong University of Science and Technology Wuhan Hubei 430074 China; ^2^ State Key Laboratory of Materials Processing and Die & Mould Technology School of Materials Science and Engineering Huazhong University of Science and Technology Wuhan Hubei 430074 China

**Keywords:** anode materials, long‐cycle life, NaPF_6_/DME, sodium‐ion batteries, TiS_2_

## Abstract

Titanium disulfide (TiS_2_) is investigated as an advanced conversion electrode for sodium (Na)‐ion batteries (NIB) in an ether‐based electrolyte (NaPF_6_/glyme (DME)). The as‐prepared TiS_2_ demonstrates a high reversible capacity of 1040 mA h g^−1^ at 0.2 A g^−1^ with the capacity contribution of 521 mA h g^−1^ in the voltage region below 1.0 V (vs Na/Na^+^), remarkable initial coulombic efficiency of 95.9% and superior rate capability of 621 mA h g^−1^ at 40 A g^−1^. The high conductivity of the Ti‐based compounds and nanosized particles generated by chemical conversion reactions could minimize the entropic barrier for the reversible conversion, resulting in high reversibility and ultrafast charge/discharge ability of the electrode. Moreover, with its strong ability to adsorb soluble polysulfide intermediates, the as‐prepared TiS_2_ electrode exhibits superior cycling stability over 9000 cycles, serving as a stable and ultra‐high capacity conversion electrode for NIBs.

## 1 Introduction

Rapid growth of the need for renewable and sustainable energy in the global energy market has inspired an urgent demand for the development of large‐scale energy storage systems with low cost and high efficiency.^1^, ^2^, ^3^ Although lithium‐ion batteries (LIBs) have successfully dominated the portable electronic market in the past decades, their large‐scale application in electric vehicles and smart grid still face significant problems due to the high cost and restricted lithium source. As the most promising alternative, Na‐ion batteries (NIBs) have recently attracted a highly revived interest owing to the widespread sodium (Na) reserves and low environmental impact.^4^, ^5^, ^6^, ^7^, ^8^ However, the larger ionic radius of the Na^+^ ion hinders the electrochemical reaction kinetics and imposes difficulties with respect to exploiting appropriate electrodes with high capacities, fast charge–discharge properties, and long‐cycle lives.

Layered transition‐metal dichalcogenides (TMDs) have been extensively investigated as energy storage materials owing to their robust structure, low cost, and environmental friendliness.^9^, ^10^, ^11^, ^12^, ^13^, ^14^, ^15^, ^16^ The unique open framework of TMDs guarantees acceptable mobility of Na^+^ insertion/desertion and diffusion. Moreover, they can deliver attractive high theoretical capacities (895 mA h g^−1^ for FeS_2,_ 558 mA h g^−1^ for CuS, etc.) through multistep reaction mechanisms, including intercalation and conversion reactions. In general, the latter reaction induces severe volume change in the electrode materials during cycling, resulting in drastic structural collapse and rapid capacity decay. By controlling the cut‐off voltage to restrain the detrimental conversion reactions, the cycling performance of TMDs could be remarkably improved.^16^ However, the inferior reversible capacity based on the intercalation reaction is far from what is expected.

The key aspect for achieving high electrochemical performances from the TMDs is to sustain the high reversibility of the conversion reactions, in which the electrolyte plays a significant role. Recent studies reveal that conventional ester‐based electrolytes are not suitable for most TMDs since the solvents would provoke side reactions with the anionic group of polysulfide. An ether‐based electrolyte with higher chemical stability is a better choice to ensure long‐term cycling stability.^12^, ^13^, ^14^, ^15^, ^16^ Furthermore, compared to intercalation or alloying reactions, the kinetics of the conversion reactions are extremely sluggish owing to the complex reaction mechanisms involving structural decomposition and reconstruction. To improve the capacity utilization and rate capability of TMDs, intensive efforts have been aimed at developing ultrafine nanoparticles so as to minimize the Na^+^ pathways and create abundant electrochemically active nanodomains for efficient and reversible reactions.^17^ However, the cycling performance of TMDs are still unsatisfactory, which can be ascribed to the dissolution of the polysulfide in the ether‐based electrolyte during the conversion reaction.^14^ Moreover, the complicated synthetic route, low compacted density, and poor initial coulombic efficiency of the nanostructured materials are not desirable for practical large‐scale applications.

To achieve significant breakthrough in the electrochemical performances of TMDs, it should be emphasized on developing strategies for suppressing the dissolution of polysulfide intermediates generated during the conversion reactions. Titanium disulfide (TiS_2_), with its ability to adsorb polysulfide, is an attractive candidate for Na storage.^18^, ^19^, ^20^, ^21^ In a recent work, Takeuchi and co‐workers have proven that TiS_2_ strongly interacts with polysulfide intermediates and can efficiently enhance the electrochemical performances of Li–S batteries by suppressing the polysulfide dissolution.^20^, ^21^ In addition, TiS_2_ is a well‐known semimetal used as the first generation cathode in metal ion batteries, with favorable intercalation kinetics.^22^, ^23^ Recently, Chaturvedi et al. prepared TiS_2_ crystalline platelets via chemical vapor transport and evaluated it as an insertion host for NIBs. It delivers a reversible capacity of 146 mAh g^−1^ at 0.1 C (1 C = 239 mA h g^−1^) with a stable cycling performance over 160 cycles.^24^ Li and co‐workers reported TiS_2_ nanoplates with a considerable Na‐storage capacity of 186 mA h g^−1^ based on the intercalation reaction, demonstrating a capacity retention of 76% over 300 cycles.^25^ To the best of our knowledge, the electrochemical conversion reaction of TiS_2_ has never been investigated in LIBs or NIBs. In fact, TiS_2_ has the lowest weight among all the TMDs, which can nearly facilitate a four‐electron transfer through electrochemical conversion of TiS_2_ to metallic Ti, with a theoretical capacity of 957 mA h g^−1^. In this contribution, we investigate the electrochemical conversion reactions of TiS_2_ in NIBs using an ether‐based electrolyte (NaPF_6_/glyme (DME)). Compared to the intercalation mechanism previously reported, the TiS_2_ electrode based on the conversion reactions demonstrate greatly improved Na‐storage performances in terms of reversible capacity, cycling stability, and rate capability. Benefiting from the high electronic conductivity of TiS_2_ and its strong affinity for the soluble polysulfide, the as‐prepared electrode demonstrates a high reversible capacity of 1040 mA h g^−1^ at 0.2 A g^−1^, strong rate capability of 621 mA h g^−1^ at 40 A g^−1^, and superior cycling stability over 9000 cycles.

## 2 Results and Discussion

**Figure 1:**
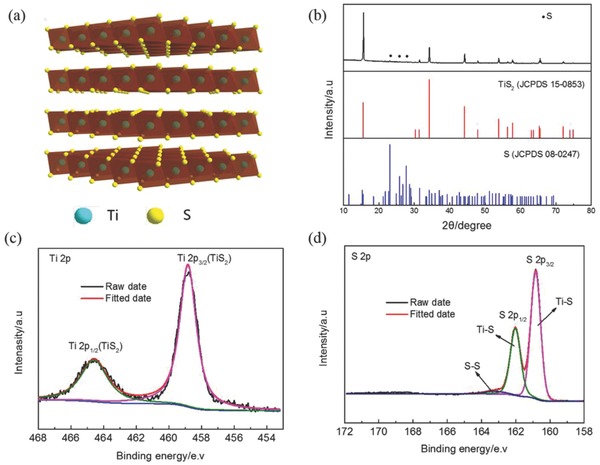
a) Crystal structure, b) XRD pattern, c) Ti 2p, and d) S 2p XPS spectra of TiS_2_.

TiS_2_ has a layered structure analogous to graphite, with the Ti ions sandwiched between two packed sulfur layers, and these are held closely together by van der Waals forces (**Figure** **1**a). The crystal structure of as‐prepared TiS_2_ is shown in Figure 1b. Except for few peaks of S which cannot be washed away by CS_2_, all the diffraction peaks can be well indexed to a hexagonal structure (standard JCPDS No. 15‐0853) with the space group P3̄ml (164).^18^, ^22^ X‐ray photoelectron spectroscopy (XPS) was carried out to investigate the elemental bonding configurations of as‐prepared TiS_2_. The high‐resolution Ti 2p XPS spectrum (Figure 1c) exhibits two peaks at 458.8 and 464.6 eV ascribed to Ti^4+^ 2p3/2 and Ti^4+^ 2p1/2, respectively.^26^ The peaks at the binding energies of 160.8, 162, and 163.3 eV in the high‐resolution S 2p spectrum indicate the presence of Ti—S and S—S covalent bonds (Figure 1d). The mass content of S is calculated to be 5.9% based on the XPS analysis and TG curve (Figure S1, Supporting Information). In the Raman spectrum (Figure S2, Supporting Information), two characteristic peaks at 234 and 333 cm^−1^ assigned to the stretching vibrations of Ti—S bond are observed, confirming the formation of TiS_2_.^27^ Furthermore, the morphology and microstructure of the as‐prepared TiS_2_ are observed by scanning and transmission electron microscopy (SEM/TEM). As shown in the SEM image (Figure S3a,b, Supporting Information), the products show a micrometer‐sized flake‐like architecture. The TEM image (Figure S3c, Supporting Information) exhibits a typical layered structure with a lateral dimension of 50 nm. The high‐resolution (HR)‐TEM image of TiS_2_ (Figure S3d, Supporting Information) shows the characteristic lattice fringes of the (101) plane with a spacing of 0.262 nm, consistent with the X‐ray diffraction (XRD) results. Energy dispersive spectroscopic mapping suggests that Ti and S species are distributed homogeneously throughout the particle (Figure S4, Supporting Information). Benefiting from the micrometer‐sized structure, as‐prepared TiS_2_ presents a relatively high tapped density of 1.55 g cm^−3^, which can be packed together densely and is favorable for practical applications.

**Figure 2:**
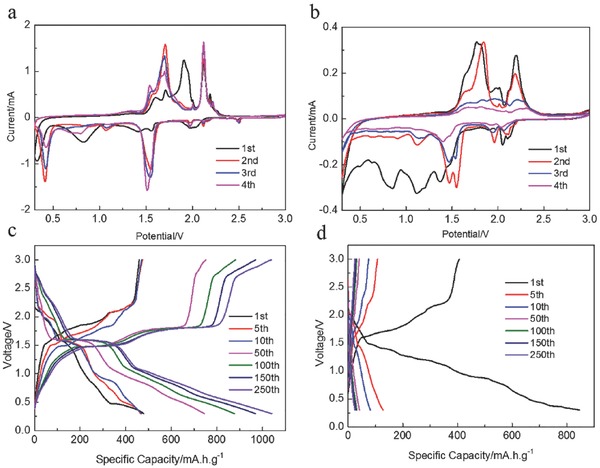
CV curves of TiS_2_ electrode in a) NaPF_6_/DME and b) NaPF_6_/EC+DEC at a scan rate of 0.1 mV s^−1^. Galvanostatic charge/discharge profiles of TiS_2_ electrode at a current density of 0.2 A g^−1^ in c) NaPF_6_/DME and d) NaPF_6_/EC+DEC.

The Na‐storage behavior of as‐prepared TiS_2_ was evaluated by both cyclic voltammetry (CV) and galvanostatic charge–discharge cycling at room temperature. **Figure** **2**a,b displays the CV curves of the as‐prepared TiS_2_ electrode in a typical ether‐based electrolyte (NaPF_6_/DME) and ester‐based electrolyte (NaPF_6_/EC+DEC) within the potential window of 0.3–3 V (vs Na/Na^+^). In the CV curves of the electrode in the ether‐based electrolyte (NaPF_6_/DME), there are several reduction peaks centered at 2.0, 1.6, 1.4, and 0.78 V in the first reduction scan, corresponding to the anodic peaks at 1.5, 1.7, 1.9, and 2.2 V, respectively. These redox peaks are ascribed to the multiple steps of intercalation and conversion reactions of layered TiS_2_. The peak located at 0.42 V, which disappears in the subsequent cycles, is ascribed to the irreversible deposition of the electrolyte and the formation of solid–electrolyte interface (SEI) films. Specifically, the CV currents increase gradually in the ensuing cycles, apparently involving an activation process of bulk TiS_2_. In contrast, as shown in Figure 2b, in the CV curves of the electrode in ester‐based electrolyte (NaPF_6_/EC+DEC), all the peaks in the first few scans visibly deteriorate, suggesting the unstable electrochemical reactions in the ester‐based electrolyte.

Figure 2c, d presents the galvanostatic charge/discharge profiles of the as‐prepared TiS_2_ electrode in different electrolytes. In the ether‐based electrolyte, TiS_2_ delivers an initial discharge/charge capacity of 478.8/459.5 mA h g^−1^ at a constant current density of 0.2 A g^−1^, corresponding to a high initial coulombic efficiency of 95.9%. Interestingly, the discharge capacity increases slowly to 1040 mA h g^−1^ and remain stable over 250 cycles. Based on the charge–discharge curves of the Ketchen black (Figure S5, Supporting Information), the capacity contribution from the carbon additives is estimated to be 49 mA h g^−1^, which is negligible compared to the high reversible capacity of TiS_2_. It is noteworthy that the reversible capacity of TiS_2_ is higher than the theoretical value of 957 mA h g^−1^. The excess reversible capacity can be ascribed to the high reversible capacity of the S impurities (1672 mA h g^−1^). It should be pointed out the critical bottleneck for the development of metal sulfide anodes is the high charge/discharge voltage, which raise difficulties for their practical applications in constructing full batteries. However, for the TiS_2_ electrode, the capacity contribution in the low voltage range (0.3–1.0 V) is estimated to be ≈521 mA h g^−1^, which is comparable to the values that reported for high‐performance carbonaceous anodes and metallic alloys,^28^, ^29^, ^30^, ^31^, ^32^ fulfilling the commercial demands for wide‐spread applications. Remarkably, the charge/discharge curves corresponding to the 250th cycle demonstrate much smaller polarization compared to the initial curves, which can be ascribed to the gradual phase transformation. In contrast, the electrode cycled in the ester‐based electrolyte (NaPF_6_/EC+DEC) demonstrates an inferior initial coulombic efficiency of 48.2%. The reversible capacity fades rapidly from 846.2 to 59 mA h g^−1^ in the first few cycles, which is in good accordance with the CV curves. The poor cycling stability in ester‐based electrolyte can be ascribed to the side reactions between the carbonate solvents and the polysulfide.

To further investigate the effect of the electrolyte, the electrochemical performances of TiS_2_ are analyzed in different ether‐based electrolytes with different Na salts and solvents (Figure S6, Supporting Information). Notably, the electrolyte cycled in all the ether‐based electrolytes not only demonstrated a much better cycling stability but also an obviously higher initial coulombic efficiency of 72.3–95.9%. Electrochemical impedance spectra (EIS) of the TiS_2_ electrodes after the first sodiation process in different electrolytes were recorded in the frequency range of 100 KHz–0.1 Hz. As shown in Figure S7 and Table S1 in the Supporting Information, the *R*_SEI_ values of the TiS_2_ electrode in NaPF_6_/DME, NaPF_6_/diglyme (DGME), NaPF_6_/tetraglyme (TGME), NaCF_3_SO_3_/DME, and NaClO_4_/DME are 4.3, 20.2, 27.4, 26.2, and 338.3 Ω, respectively, whereas that in NaPF_6_/EC+DEC is 523.6 Ω, indicating the formation of a thinner SEI film and better ionic conduction of the ether‐based electrolyte. In addition, the charge transfer resistance (*R*_ct_) of the electrode cycled in the ether‐based electrolytes (*R*_ct_ = 12.7–32.7 Ω) is much lower than that cycled in the ester‐based electrolyte (*R*_ct_ = 50.5 Ω), suggesting that the ether‐based electrolyte can effectively improve the electronic transfer kinetics. Apart from choosing an appropriate electrolyte, controlling the terminal voltage to an appropriate range is also crucial for enhancing the electrochemical performances of TMDs. For achieving optimized electrochemical performance, the cycling performance of the as‐prepared electrode was evaluated over various voltage ranges, as shown in Figure S8 in the Supporting Information. When the voltage range is narrowed to 0.5–3.0 and 1.0–3.0 V, TiS_2_ exhibits compromised reversible capacities of 469 and 327 mA h g^−1^. However, when the voltage range is extended to 0.005–3.0 V, a serious capacity fading could be observed after 400 cycles. As a result, controlling the discharge voltage to 0.3 V provides the best electrochemical performance in terms of the reversible capacity and cycling stability.

**Figure 3:**
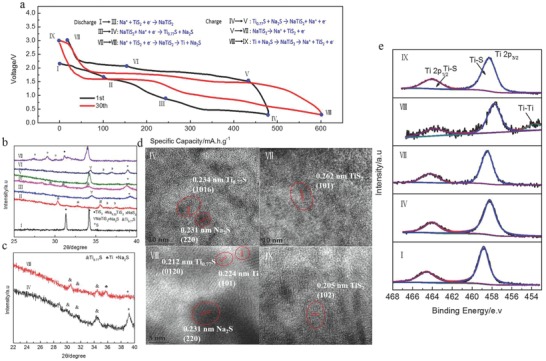
Ex situ characterization of TiS_2_ electrodes at different charge–discharge states: a) Charge/discharge profiles of the initial and the 30th cycle, b,c) XRD patterns, d) HRTEM images,and e) XPS spectra. The marks (I–IX) in (b)–(e) correspond to the marks in (a).

It is noteworthy that the reversible capacity increases continuously in the first several hundreds of cycles. To unveil the underlying reason for this continuous increase in capacity and gain insights into the reaction mechanism, ex situ XRD, TEM, and XPS characterizations were carried out to investigate the evolution of the electrode structure during cycling. **Figure** **3**a presents the typical charge/discharge profiles of the TiS_2_ electrode for the initial and 30th cycle. During the first discharge process (I→II→III→IV), the XRD patterns of the electrode reveal the phase transformation from TiS_2_ to Na_0.22_TiS_2_ and NaTiS_2_ (Figure 3b), indicating the step‐wise intercalation of Na^+^ into the TiS_2_, which is consistent with the results reported previously.^25^ Upon further discharge to 0.3 V, the characteristic peaks of layered Na‐Ti‐S gradually disappear and peaks at 30°, 30.9°, and 34.2° appear, which can be attributed to the structural transformation of layered Na‐Ti‐S to Ti_0.77_S. The HR‐TEM image confirms the clear lattice fringes with a spacing of 0.234 nm at the state IV, corresponding to the (101) crystal planes of Ti_0.77_S (Figure 3d). Moreover, the binding energy of Ti 2p2/3 shifts from 458.9 to 458.3 eV in the high‐resolution XPS spectrum of the electrode (Figure 3e), indicating the reduction of TiS_2_. When recharged back to 3.0 V, the electrode reverts to its original state through the structural evolution generalized as Ti_0.77_S → NaTiS_2_ → TiS_2_. Moreover, the charge and discharge products obtained after 30 cycles were also investigated. As shown in Figure 3c,e, the XRD and XPS results reveal the coexistence of Ti_0.77_S and metallic Ti at the discharged state corresponding to the 30th cycle. Compared to the initial cycle, the electrode is reduced to a lower valence state in the subsequent cycles, resulting in higher electrochemical utilization and reversible capacity. Density functional theory (DFT) calculations confirm that the reduced products, NaTiS_2_ and Ti_0.77_S exhibit much higher electronic conductivity than pristine TiS_2_ (Figure S9, Supporting Information). As a result, the formation of NaTiS_2_, Ti_0.77_S, and metallic Ti can provide a rapid kinetic pathway for the electronic transport in the interior of the electrode, leading to smaller polarization and higher electrochemical utilization of the active materials.

In addition to the structural transformation, morphological changes in the electrode were observed by ex situ SEM imaging. As shown in Figure S10 in the Supporting Information, the microsized TiS_2_ electrode has pulverized to nanocrystals during the conversion reactions which involve repeated decomposition and reconstruction of the structure. For the bulk electrode, chemical conversion reactions are restricted to the surface of the particle owing to kinetic and thermodynamic limitations, resulting in inferior reversible capacities in the initial few cycles. When pulverized to nanosized particles, the nanoscale reaction domains can significantly overcome the kinetics and thermodynamic constraints and afford a rapid kinetic pathway for complete electrochemical transformations, leading to complete transformation from TiS_2_ to metallic Ti and Na_2_S (Equations S1 and S2, Supporting Information).

**Figure 4:**
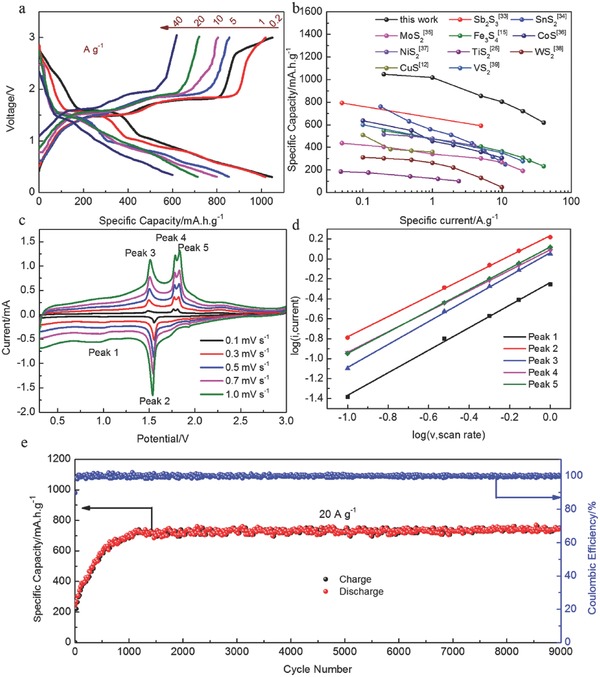
Electrochemical performances of the TiS_2_ electrode. a) Charge/discharge profiles at various current rates ranging from 0.2 to 40 A g^−1^, b) comparison of the as‐prepared TiS_2_ with other sulfide materials of sodium ion batteries, c) CV curves of as‐prepared TiS_2_ after 100 cycles at different scan rates ranging from 0.1 to 1.0 mV s^−1^, d) log(i) versus log(v) plots, and e) long‐term cycling performance.

The rate capability and long‐term cycling stability of a battery are the key characteristics for their practical application. **Figure** **4**a exhibits the charge/discharge curves of the Na/TiS_2_ batteries at different current densities ranging from 0.1 to 40 A g^−1^. As‐prepared TiS_2_ exhibits reversible capabilities of 1047, 1017, 853, 799, and 713 mA h g^−1^ at the current densities of 0.2, 1.0, 5, 10, and 20 A g^−1^, respectively. More encouragingly, upon increasing the current density to 40 A g^−1^, a reversible capacity of 621 mA h g^−1^ could still be maintained, indicating that the charge/discharge process can go to completion within 1 min. Compared to other TMDs, this TiS_2_ demonstrates a remarkably fast charge–discharge capability, as summarized in Figure 4b.^12^, ^15^, ^25^, ^33^, ^34^, ^35^, ^36^, ^37^, ^38^, ^39^ CV measurements of the TiS_2_ electrodes at different scanning rates were carried out to explain the high rate capability and cycling stability (Figure 4c,d). The charge storage mechanism always involves non‐Faradaic and Faradaic processes. The capacitive effect of the battery system is calculated according to Equations S3 and S4 in the Supporting Information. The b‐value is calculated to be ≈1 for all the peaks, which is a significant characteristic indicating that the Na/TiS_2_ batteries are mainly governed by capacitance. The capacitive process and high conductivity enables a fast charge/discharge process, resulting in superior rate performance and an extended cycling life. Furthermore, the formation of highly conductive intermediate products (NaTiS_2_, Ti_0.77_S, etc.) is also significantly beneficial for the fast charge–discharge ability.

The long‐term cycling stability of the Na/TiS_2_ battery was further evaluated at different current densities ranging from 5 to 40 A g^−1^. As shown in Figure S11 and S12 in the Supporting Information, after the activation process, the TiS_2_ electrode remains stable over 4000 cycles at different current densities ranging from 5 to 40 A g^−1^. More encouragingly, an impressive long‐term cycling performance is achieved at the current density of 20 A g^−1^. As shown in Figure 4e, TiS_2_ can deliver a capacity of 740 mA h g^−1^ and remain stable over 9000 cycles. The coulombic efficiency rapidly rise up to 99% in the first few cycles, indicating the high electrochemical reversibility of the TiS_2_ electrode at such ultra‐high current density. Such excellent long‐term cycling stability has been rarely reported in the previous literatures. Apart from the high chemical stability of the ether‐based electrolyte, the impressive long‐term cycling stability can also be ascribed to the strong chemisorption of TiS_2_ on the polysulfide (Figure S13, Supporting Information). For most TMDs, the polysulfide generated during the long‐term cycling dissolves in the ether‐based electrolyte, leading to a poor cycling life. However, the TiS_2_ electrode can entrap the polysulfides on its surface (Figure S14, Supporting Information), leading to a stable chemical composition of the electrode.

## 3 Conclusion

In summary, TiS_2_ is investigated as a conversion electrode for NIB in an ether‐based electrolyte. Benefiting from the high conductivity and strong surface affinity to the polysulfide intermediates, as‐prepared TiS_2_ delivers a high reversible capacity of 1040 mA h g^−1^ at 0.2 A g^−1^, remarkable initial columbic efficiency of 95.9%, strong rate capability of 621 mA h g^−1^ at 40 A g^−1^, and superior cycling stability over 9000 cycles. In addition, the nanosized particles generated during the conversion reaction can minimize the entropic barrier for the reversible conversion reaction, leading to high electrochemical utilization and superior cycling stability. This work may pave the way for the development of high‐capacity conversion materials with stable cycling for prospective energy storage applications.

## 4 Experimental Section

*Materials Synthesis*: TiS_2_ samples were synthesized by a solid‐phase sintering method under vacuum. Typically, 0.1 g titanium and 0.3 g sulfur were mixed thoroughly, and then the mixture was heated under vacuum at 660 °C for 7 days. After cooling down, the brass power was centrifuged and washed with carbon disulfide for more than ten times to remove excess sulfur. Then the samples were dried at 80 °C under vacuum for 10 h to obtain the target material of TiS_2_.

*Materials Characterization*: The structure information was investigated by XRD (X'Pert PRO Diffractometer). The morphologies were observed by SEM (FEI Sirion 200) and TEM (JEOL JME‐2010F). Raman spectra were collected on a LabRAM HR800 Raman system at a laser wavelength of 532 nm. XPS (AXIS‐ULTRA DLD spectrometer) was carried out to investigate the elemental bonding configurations. S content was investigated by TG‐DSC (Netzsch STA 449 F5) in an air atmosphere at a ramping rate of 10 °C min^−1^.

*Electrochemical Measurements*: Except for using 1 m NaPF_6_/DME as the electrolyte, the working electrode and coin cells were prepared as reported previously and the active material loading was ≈2.2–2.5 mg cm^−2^.^37^ CV measurements and EIS were performed on a CHI 660D electrochemical workstation. The galvanostatic charge/discharge tests were performed on a LAND battery test system.

## Conflict of Interest

The authors declare no conflict of interest.

## Supporting information

Supplementary
