# Supplementary material for: TiS2 as an Advanced Conversion Electrode for Sodium‐Ion Batteries with Ultra‐High Capacity and Long‐Cycle Life
Source: Adv Sci (Weinh). 2018 Sep 15;5(11):1801021. doi: 10.1002/advs.201801021 (PMC6247063; doi:10.1002/advs.201801021)
Supplement: Supplementary file 1 — Supplementary [file ADVS-5-1801021-s001.pdf]

Copyright WILEY-VCH Verlag GmbH & Co. KGaA, 69469 Weinheim, Germany, 2018.

Supporting Information

for *Adv. Sci.,* DOI: 10.1002/advs.201801021

TiS_2 as an Advanced Conversion Electrode for Sodium-Ion Batteries with Ultra-High Capacity and Long-Cycle Life

Hongwei Tao, Min Zhou, Ruxing Wang, Kangli Wang, Shijie Cheng, and Kai Jiang**

Copyright WILEY-VCH Verlag GmbH & Co. KGaA, 69469 Weinheim, Germany, 2018.

Supporting Information

TiS_2 as an Advanced Conversion Electrode Material for Na-ion Batteries with Ultra-High Capacity and Long-Cycle Life

Hongwei Tao, Min Zhou, Ruxing Wang, Kangli Wang, Shijie Cheng, Kai Jiang**

1. TG curve of TiS_2

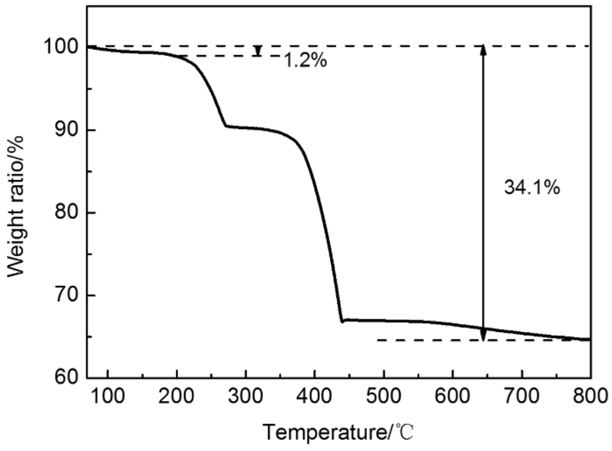

Figure S1. TG curve of TiS_2

The content of impurity S is investigated by TG analysis as shown in Figure S1. In this figure, the initial weight loss under 200 °C can be attributed to the evaporation of water. The weight loss in the voltage range of 200-800 °C can be ascribed to the oxidation of S and TiS_2. By calculation, the S content in TiS_2 is estimated to be 5.9%.

2. Raman spectrum of TiS_2

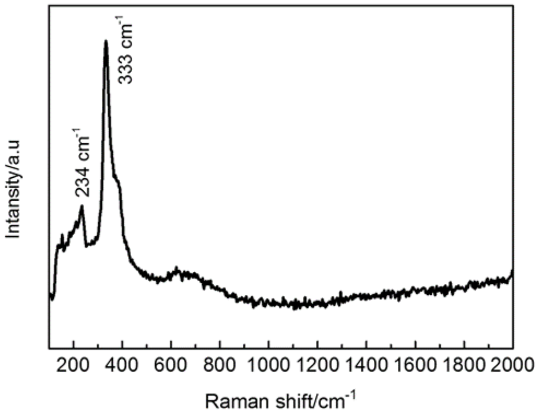

Figure S2. Raman spectrum of TiS_2

3. Scanning and transmission electron microscopy (SEM/TEM) images of TiS_2

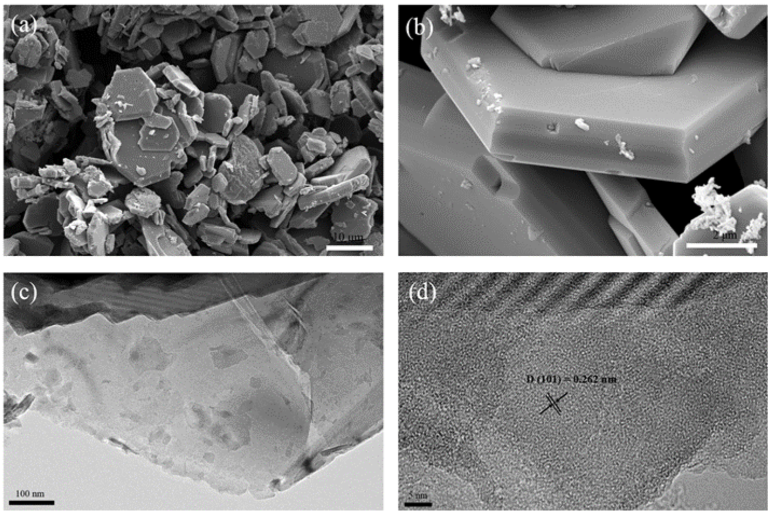

Figure S3. (a-b) SEM and (c-d) TEM images of TiS_2.

4. EDS mappings of TiS_2

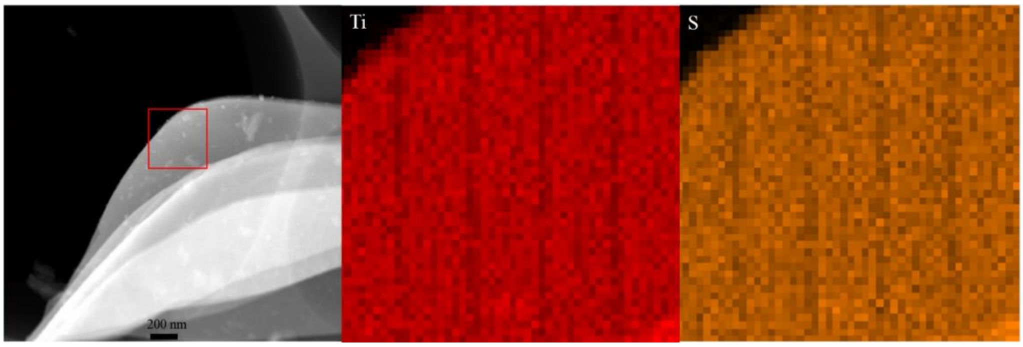

Figure S4. EDS mappings of as-prepared TiS_2.

5. Electrochemical performances of the carbon addictive

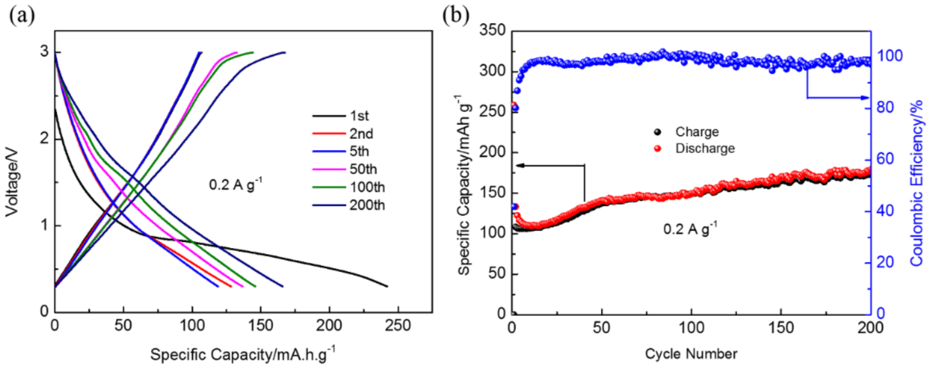

Figure S5. The charge-discharge profiles (a) and cycling performance (b) of Ketchen black in 1 M $NaPF_6$/DME. Current density: 0.2 A g^{-1}; Voltage range: 0.3 ~ 3 V

6. Electrochemical performance of TiS_2 in different ether-based electrolytes with different Na salts and solvents

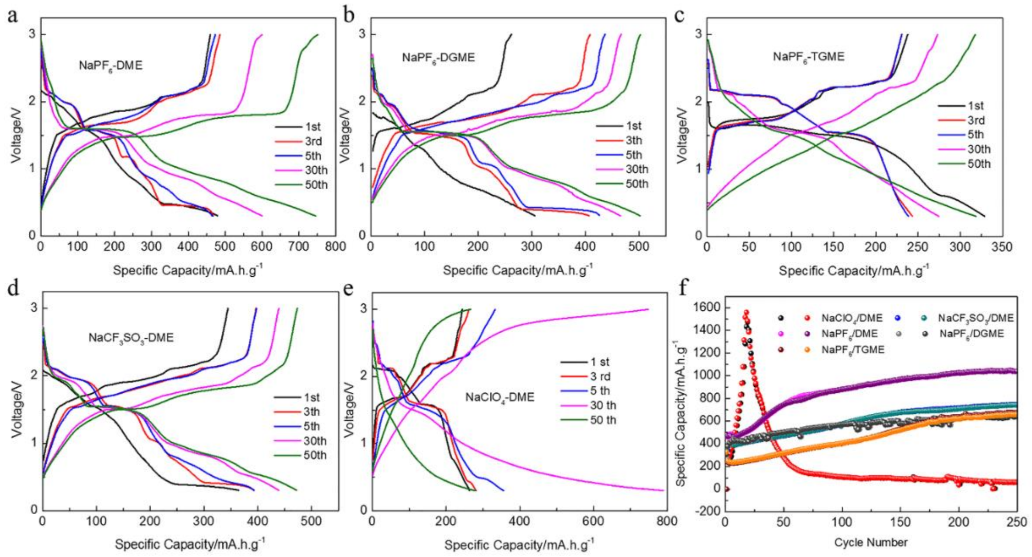

Figure S6. Charge-discharge curves of TiS_2 at 1 A g^{-1} in (a) $NaPF_6$/DME, (b) $NaPF_6$/DGME, (c) $NaPF_6$/TGME, (d) $NaCF_3SO_3$/DME, and (e) $NaClO_4$/DME. (f) Cycling performance of TiS_2 in different ether-based electrolytes at 1 A g^{-1}.

The electrochemical performances of TiS_2 in different ether-based electrolytes with different Na salts and solvents are shown in Figure S6. In this figure, the initial Coulombic efficiency of TiS_2 electrodes using $NaPF_6$/DME, $NaPF_6$/DGME, $NaPF_6$/TGME, $NaCF_3SO_3$/DME, and $NaClO_4$/DME at the current of 1 A g^{-1} are 95.9, 85.6, 72.3, 94.2, and 92.5%, respectively, and the reversible capacities remain stable at 1040.2, 656.3, 678.7, 746.5, and 58 mA h g^{-1}, respectively, after 250 cycles, indicating that the TiS_2 electrode in the $NaPF_6$/DME electrolyte shows optimal electrochemical performance.

7. Electrochemical impedance spectra (EIS) of the TiS_2 in different electrolytes

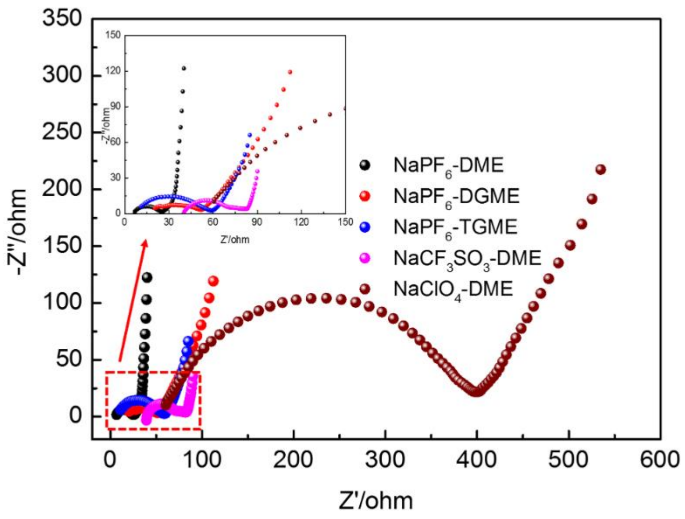

Figure S7. EIS of TiS_2 after 250 cycles in different electrolytes. The inset shows the corresponding equivalent circuit model.

Table S1. Fitting results for the Nyquist plots obtained using the equivalent circuit.

Samples	R_S(Ω)	R_{SEI} (Ω)	CPE_S(F)	R_{ct}(Ω)	CPE_{dl}(F)	Chi-Squared
$NaPF_6$-DME	6.6	4.3	1.4×10^{-6}	12.7	2.0×10^{-5}	1.1×10^{-3}
$NaPF_6$-DGME	15	20.2	1.9×10^{-8}	13.3	2.1×10^{-4}	8.1×10^{-4}
$NaPF_6$-TGME	7.8	27.4	4.7×10^{-6}	14.7	1.9×10^{-4}	2.7×10^{-4}
$NaCF_3SO_3$-	40.1	26.5	1.6×10^{-5}	13.2	3.0×10^{-3}	2.3×10^{-3}

DME						
$NaClO_4$-DME	55	338.3	7.9×10^{-6}	32.7	1.5×10^{-7}	1.5×10^{-4}
$NaPF_6$-EC-DEC	2.8	523.6	4.5×10^{-5}	50.5	1.4×10^{-5}	8.4×10^{-4}

8. Cycling performance of TiS_2 over various voltage ranges

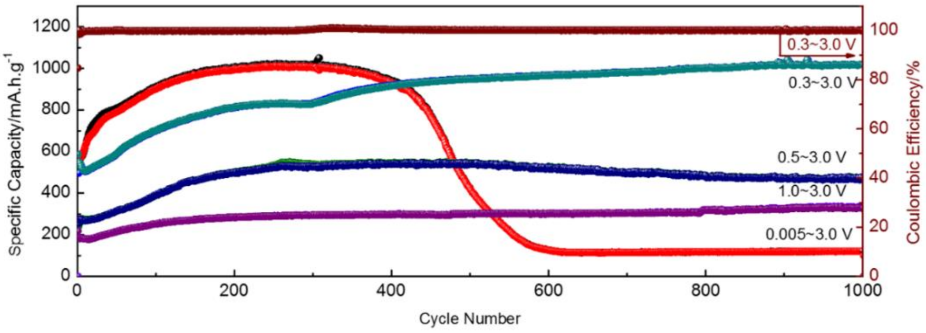

Figure S8. Cycling performance of TiS_2 over various voltage ranges.

9. Density function theory (DFT) calculations for TiS_2, $NaTiS_2$, and $Ti_{0.77}S$

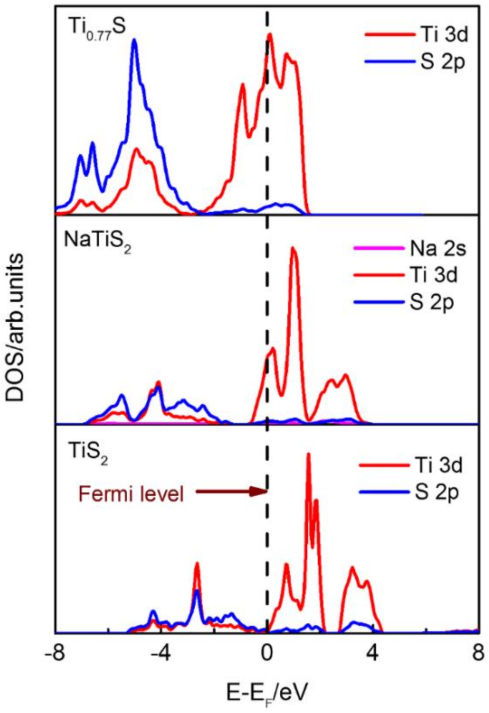

Figure S9. The partial density of states of TiS_2, $NaTiS_2$, and $Ti_{0.77}S$. The dotted line represents the Fermi level.

Theoretical calculations. DFT calculations were performed using the DACAPO package[1] within PBE[2] describing the exchange and correlation functional. Ultrasoft pseudopotentials[3] were used to model the core electrons, while plane waves with a kinetic energy cutoff of 480 eV was chosen to expand the valence electron wavefunction throughout. Single point energy calculations were performed for the primitive cell of TiS_2, $Ti_{0.77}S$, and $NaTiS_2$ based on their respective experimental geometries; the corresponding Brillouin zones were sampled using Monkhorst-Pack[4] meshes with sizes of 12 × 12 × 8, 12 × 12 × 2, and 12 × 12 × 1, respectively.

Figure S9 shows the partial density of states (PDOS) of TiS_2, $NaTiS_2$, and $Ti_{0.77}S$. The PDOS of TiS_2 presents an obvious gap corresponding to the Fermi level, indicating the semiconducting nature of TiS_2. In comparison with TiS_2, both $NaTiS_2$ and $Ti_{0.77}S$ show obvious states in the Fermi level, suggesting the metallicity and increased electronic conductivity of these two materials. The metallicity can be ascribed to the p orbitals of the S atoms and d orbitals of the Ti atoms. The metallicity of $NaTiS_2$ and $Ti_{0.77}S$ can significantly enhance the electronic conductivity, resulting in the excellent electrochemical performance of the electrode.

10. Scanning electron micrographs (SEM) of the TiS_2 electrode after different cycles in the fully charged state

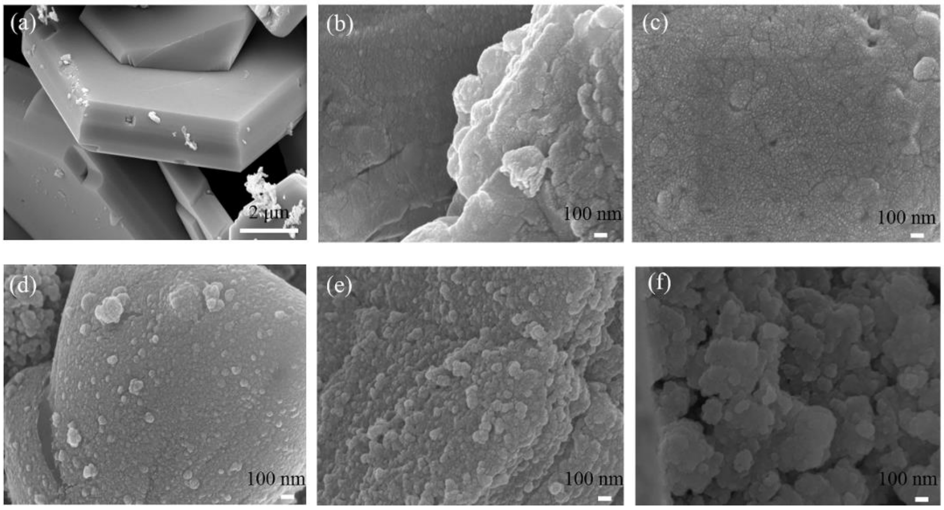

Figure S10. SEM of the TiS_2 electrode after different cycles, in the fully charged state, (a) pristine electrode, and after (b) 1 cycle, (c) 10 cycles, (d) 100 cycles, (e) 200 cycles, and (f) 700 cycles.

11. The reaction mechanism of TiS_2

$$Na^+ + TiS_2 + e^- \rightarrow NaTiS_2 \quad \text{(S1)}$$

$$3\ Na^+ + NaTiS_2 + 3\ e^- \rightarrow 3\ Ti + 2\ Na_2S \quad \text{(S2)}$$

12. The capacitive behavior of TiS_2

In fact, non-Faradaic and Faradaic behaviors control the entire cycle process. The contribution of the reaction behavior could be analyzed according to Equations S3 and S4:

$$i = av^b \quad \text{(S3)}$$

$$\log(i) = \text{b}\log(v) + \log(a) \quad \text{(S4)}$$

where, i is the peak current, v is the scan rate, and a and b are constants. In particular, the electrochemical behavior of the cycle process is determined by the b value. When the b value approaches 0.5, it indicates that the electrochemical reaction is dominated by ionic diffusion, whereas, when it approaches 1, the capacitive behavior dominates the total process. Figure 4d shows the liner fitting curves of log(i) and log(v) at different potentials. Using this figure, the

b-value of Peak 1, Peak 2, Peak 3, Peak 4, and Peak 5 are calculated to be 1.13, 1.01, 1.15, 1.02, and 1.07, respectively, indicating that the entire electrochemical reaction of the TiS_2 electrode is dominated by capacitive behavior.

13. Long term cycling performance of the TiS_2 electrode at different current densities

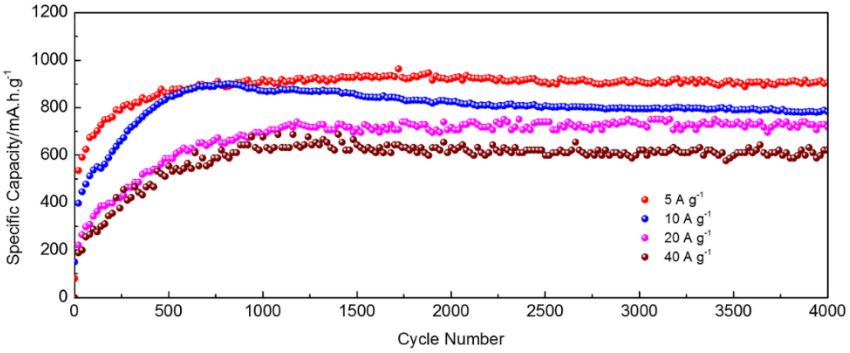

Figure S11. Long term cycling performance of the TiS_2 electrode at different current densities ranging from 5 to 40 A g^{-1}.

As shown in Figure S11, the TiS_2 electrode is stable over 4000 cycles after activation and delivers reversible capacities of 905.1, 783.3, 729.1, and 610.3 mA h g^{-1} at the current densities of 5, 10, 20, and 40 A g^{-1}, respectively.

14. Na storage performances of the TiS_2 at 40 A g^{-1}

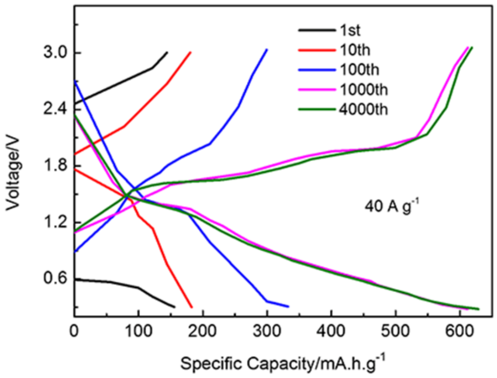

Figure S12. Charge-discharge profiles of the TiS_2 in $NaPF_6$/DME at 40 A g^{-1}.

15. The adsorption capability of as-prepared TiS_2

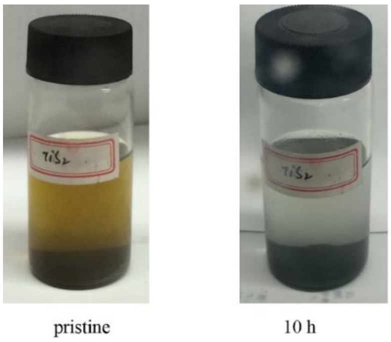

Figure S13. Sealed vials of a sodium polysulfide solution (Na_2S_6 dissolved in DME solvent) containing TiS_2 powder: (a) Pristine and (b) after 10 h.

The adsorption ability of as-prepared TiS_2 is shown in Figure S13. In this figure, the pristine color of the Na_2S_6 solution after mixing with TiS_2 is dark yellow. However, the solution became almost colorless after 10 h, indicating the strong chemisorption of TiS_2 on the polysulfide.

16. S 2p XPS spectra of TiS_2 electrode after 30 cycles in the fully discharged state

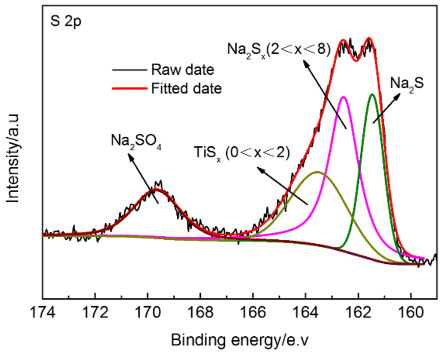

Figure S14. S 2p XPS spectra of TiS_2 electrode after 30 cycles in the fully discharged state

Figure S14 shows the XPS S 2p spectra of TiS_2 electrode at the fully discharged state after 30 cycles. In the high-resolution S 2p XPS spectra, four peaks are observed at 161.5, 162.6, 163.5 and 169.7 eV, corresponding to Na_2S, polysulfides (Na_2S_x ($2<x<8$)), low valence state of titanium sulfide (TiS_x ($0<x<2$)) and Na_2SO_4, respectively. The existence of polysulfides on electrode further confirm that the TiS_2 electrode can entrap the polysulfides on its surface.

References

[1] https://wiki.fysik.dtu.dk/dacapo.

[2] D. Vanderbilt, *Phys. Rev. B* **1990**, *41*, 7892.

[3] J. P. Perdew, K. Burke, M. Ernzerhof, *Phys. rev. lett.* **1996**, *77*, 3865.

[4] H. J. Monkhorst, J. D. Pack, *Phys. rev. B* **1976**, *13*, 5188.
